# Age at diagnosis, lifestyle factors, and risk of mortality: a city-wide cohort study of cancer survivors

**DOI:** 10.1007/s00520-026-10325-6

**Published:** 2026-01-21

**Authors:** Xiaoyi Lin, Huan Xu, Suixiang Wang, Yuanyuan Chen, Ke Li, Boheng Liang, Lin Xu

**Affiliations:** 1 School of Public Health, Sun Yat-Sen University, No. 74 Zhongshan 2nd Road, Guangzhou, 510080 Guangdong China; 2 Guangzhou Center for Disease Control and Prevention, No.1 Qide Road, Baiyun District, Guangzhou, 510403 China; 3 School of Public Health, the University of Hong Kong, Hong Kong, China; 4 Department of Applied Health Sciences, School of Health Sciences, College of Medicine and Health, University of Birmingham, Birmingham, UK; 5 Greater Bay Area Public Health Research Collaboration, Guangzhou, China

**Keywords:** Cancer survivors, Diagnostic age, Lifestyle, Mortality, Cohort study

## Abstract

**Background:**

The growing global cancer burden highlights the urgent need to improve long-term outcomes among cancer survivors. Age-related biological changes may modify the associations between lifestyle factors and mortality, yet the joint effects of diagnostic age and lifestyle factors remain poorly understood.

**Methods:**

Cancer survivors diagnosed between 2010 and 2018 in Guangzhou were followed up until 2023. Associations of lifestyle factors with all-cause mortality risk were examined, stratified by early-onset (< 50 years) and late-onset (≥ 50 years) cancers. Interactions were evaluated on multiplicative and additive scales.

**Results:**

Among 22,079 cancer survivors, 10,839 deaths occurred during a median follow-up of 4.14 years. An antagonistic interaction of diagnostic age with physical activity on all-cause mortality risk was observed. Compared with inactivity, physical activity (≥ 150 min/week) was associated with a 15% lower risk of all-cause mortality (restricted mean survival time [RMST] difference: 0.29 years) in early-onset survivors and a 23% lower risk in late-onset survivors (RMST difference: 0.69 years). A synergistic interaction of diagnostic age with sleep duration was observed. Compared with 7 h/day, short sleep duration (≤ 5 h/day) was associated with a higher mortality risk (RMST difference: −0.43 years) in early-onset survivors, while 8 h/day was associated with a lower risk in late-onset survivors (RMST difference: 0.13 years).

**Conclusions:**

Sufficient physical activity and sleep duration were significantly associated with improved survival, with their effects varying by diagnostic age. These findings underscore the importance of tailored lifestyle management strategies for early-/late-onset cancer survivors to mitigate mortality burden.

**Supplementary Information:**

The online version contains supplementary material available at 10.1007/s00520-026-10325-6.

## Introduction

The number of cancer survivors has increased globally [1], driven by earlier detection, advances in treatment, and population aging [2]. In China, an estimated 4.82 million new cancer cases occurred in 2022 [3], accounting for approximately one-fourth of all new cases worldwide [1]. The global burden of cancer mortality is projected to increase by about 90% between 2022 and 2050 [4]. This growing burden underscores the urgent need to identify interventions to improve the long-term health of cancer survivors. It was estimated that 45% of cancer deaths could be attributable to modifiable risk factors [5], including cigarette smoking [6], alcohol use [6], insufficient physical activity (PA) [7, 8], and sleep problems [9]. Despite these recognized factors, cancer survivors often have low awareness and adherence to health interventions due to additional obstacles.

Unhealthy lifestyles can accelerate immunosenescence, a process marked by immune cell senescence, chronic inflammation, and genetic and epigenetic alterations, thereby facilitating cancer initiation and progression [10]. Age functions as a biological amplifier of immunosenescence and may therefore modify the association between lifestyle factors and cancer mortality [11]. Moreover, older adults often receive less intensive treatment due to comorbidities, which may contribute to poorer survival outcomes [12]. In this context, potential lifestyle-related benefits become particularly important in this group. Our previous study found that the adverse impact of unhealthy lifestyles on mortality risk appeared to be more pronounced in younger cancer survivors [13]. Conversely, findings from a comparative risk assessment conducted in China showed that the burden of cancer mortality attributable to unhealthy lifestyles was more substantial in older cancer survivors [5]. However, the extent to which the effect of each lifestyle is modified by age at diagnosis remains to be elucidated.

In the present study, using data from a city-wide prospective cohort of cancer survivors, we examined the joint associations of diagnostic age and lifestyle factors with mortality risk to provide tailored lifestyle interventions for early-/late-onset cancer survivors.

## Methods

### Study design

This study included participants who were first diagnosed with cancer between 2010 and 2018, as recorded in the Guangzhou Cancer Registry (GCR) of the Guangzhou Center for Disease Control and Prevention (GZCDC). Details of the GCR have been reported elsewhere [13, 14]. The surveillance and follow-up system of the GCR was established in 2010 [15], which covered residents across all 11 districts of Guangzhou, the provincial capital of Guangdong in southern China. The establishment of the GCR was approved by the Ministry of Finance of the People’s Republic of China, National Health Commission of the People’s Republic of China, Guangzhou Municipal Finance Bureau, and Guangzhou Municipal Health Commission. Ethical approval of this study was obtained from the ethical committee of the GZCDC.

In the GCR, cancer survivors were referred to local primary care centers 1 month after their hospital discharge following their initial cancer diagnosis. At local primary care centers, they participated in surveys using validated brief questionnaires. Information on demographic characteristics, lifestyles, and medical history, as well as anthropometric parameters including height and weight, were collected. Comprehensive cancer-specific information including pathological or clinical diagnoses, dates of diagnosis, tumor-node-metastasis (TNM) staging, and treatment histories was obtained through linkage with electronic medical records from respective hospitals. The classification of cancer types was coded according to the 10th Revision of the International Classification of Diseases (ICD-10) by trained clinical coding officers. This study included all cancer types across 15 major cancer sites [16].

### Exposures

The classification of cancer onset was determined by the age at initial diagnosis of the primary cancer. Cancers identified in participants younger than 50 years were categorized as early-onset, while those diagnosed at 50 years or older were classified as late-onset [17, 18]. Smoking status was defined as current if an individual smoked at least one cigarette/day in the past 30 days. Participants were considered never or former smokers if they had never smoked or quit smoking. Current alcohol use was defined as at least 10 g of alcohol consumption per day in the past 30 days, while never or former alcohol use was defined as abstaining from or having discontinued alcohol use. The average time spent in PA of any intensity and sleep in the past 30 days was investigated, and fractional responses were coded as rounded integers. To enable comparison with previous studies, PA was categorized as without any PA (inactive), with PA more than 0 min/week but less than 150 min/week (insufficiently active), and with PA of 150 min/week or more (active) based on the Physical Activity Guidelines for Americans [8, 19]. Sleep duration was categorized into five groups (≤ 5, 6, 7, 8, ≥ 9 h/day) following the National Sleep Foundation’s recommendations [9, 20].

### Outcomes

Until June 30, 2023, mortality data, including dates and primary causes of death, were obtained through record linkage with the death registry of the GZCDC. The primary endpoint was all-cause mortality, and the secondary endpoint was cause-specific mortality. Overall survival and cause-specific survival periods were calculated from the date of cancer diagnosis to the date of death, attributable to any cause or to specific causes, respectively. Cancer-specific mortality was identified using codes C00-C97 in accordance with the ICD-10, and cardiovascular disease (CVD)-specific mortality using codes I00-I99, with the exception of I26 and I27.

### Covariates

The analysis accounted for potential confounders, including sex, TNM staging of primary cancer, treatment modalities (surgery, chemotherapy, or radiotherapy), diagnosis of multiple primary cancers, family history of cancer, body mass index (BMI), education level (primary or below, secondary, college or above), and employment status. The TNM classification, denoting the extent of primary tumor (T), involvement of regional lymph nodes (N), and presence of distant metastasis (M), was determined based on pathological or clinical findings according to the most recent edition of the TNM classification manuals issued by the American Joint Committee on Cancer (AJCC) or the International Union Against Cancer (UICC). BMI was calculated by dividing the weight in kilograms by the square of the height in meters.

### Statistical analysis

Baseline characteristics by survival status were compared using Student’s *t*-tests for continuous variables, Wilcoxon rank-sum tests for ordinal variables, and Pearson’s chi-squared test for categorical variables. The associations of age and lifestyle factors with mortality risk were examined using multivariable Cox proportional hazards regression models. Hierarchical models were used for adjustment, including the minimally adjusted model accounting for age, sex, T stage, N stage, and M stage, and a fully adjusted model that further accounted for treatment (surgery, chemotherapy, radiotherapy), the presence of multiple primary cancers, family history of cancer, BMI, education, and employment status. Hazard ratios (HRs) and 95% confidence intervals (CIs) were estimated in both minimally and fully adjusted models. Additional analyses were conducted for PA and sleep durations, estimating HRs per 60-min increment in PA and per 1-h increment in sleep duration. The Schoenfeld residual test was used to assess the proportional hazards assumption, and no significant deviations were indicated. Given the limited follow-up and the right-censored survival data, the restricted mean survival time (RMST) was also calculated using the Kaplan-Meier estimator to assess the univariate association. The RMST, reflecting the average survival time within a 5-year period, serves as an estimate of 5-year life expectancy [21]. The difference between two RMSTs was defined as the difference in life expectancy [22, 23].

To examine the joint associations of age at diagnosis and lifestyle factors with mortality risk, we estimated both multiplicative and additive interactions. The multiplicative interactions were expressed as the effects of the interaction terms between lifestyle factors and each standard deviation (SD) increase in age at diagnosis. The additive interactions were characterized by calculating the relative excess risk due to interaction (RERI), the attributable proportion (AP) due to the interaction, and the synergy index (SI), with 95% CIs for these measures derived via bootstrap methods [24]. For significant interactions, the associations of lifestyle factors with mortality risk were examined by subgroups of early-/late-onset cancer survivors. In sensitivity analysis, we extended the examination to include associations in cancer survivors diagnosed with common types of cancer, as well as those with and without metastasis or low BMI status (< 18.5 kg/m^2^ for participants aged < 70 and < 20 kg/m^2^ for those aged ≥ 70) [25]. To reduce the possibility of reverse causation bias, subgroup analyses excluding deaths occurring within 2 months post-diagnosis were conducted [26]. Two-sided *P*-value < 0.05 was considered statistically significant. All statistical analyses were performed using R software (version 4.3.1).

## Results

Of 22,759 cancer survivors registered in the GCR between 2010 and 2018, those with missing information on PA time (*n* = 34), sleep duration (*n* = 6), and height and weight (*n* = 640) were excluded. Consequently, 22,079 cancer survivors were included, with 49.53% being men and a median age of 60 years. The distribution of cancer sites is shown in Supplementary Table 1, with the majority of cases involving the digestive and respiratory systems. During a median follow-up of 4.14 years (standard deviation = 2.10 years) with 81,124 person-years, 10,839 deaths occurred. Of these, 9054 (83.53%) were attributed to cancer and 585 (5.40%) were due to CVD.

Table 1 shows that cancer survivors who died during the follow-up period were older, predominantly men, and presented with lower BMI and more advanced TNM stages (all *P*-values < 0.001). They were less likely to receive surgery, more likely to undergo chemotherapy and radiotherapy, more likely to develop multiple primary cancers, had a higher proportion of current smokers and drinkers, and had lower PA levels (all *P*-values < 0.001).

**Table 1:** Baseline characteristics of 22,079 cancer survivors (2010–2018)

Characteristics	Death		*P*
	No ***N*** = 11,240	Yes ***N*** = 10,839	
Age, years, mean (SD)^a^	55.61 (13.24)	64.99 (13.06)	< 0.001
Sex, men, *N* (%)	4211 (37%)	6725 (62%)	< 0.001
Body mass index, kg/m^2^, mean (SD)^a^	22.41 (2.83)	21.81 (3.36)	< 0.001
Medical history			
Tumor-node-metastasis (TNM) stage			
Tumor (T) stage, *N* (%)			< 0.001
T0	120 (1.1%)	729 (6.7%)	
T1	3738 (33%)	1339 (12%)	
T2	3346 (30%)	2241 (21%)	
T3	2417 (22%)	2585 (24%)	
T4	1619 (14%)	3945 (36%)	
Lymph node (N) stage, *N* (%)			< 0.001
N0	6667 (59%)	3789 (35%)	
N1	2657 (24%)	2613 (24%)	
N2	1375 (12%)	2721 (25%)	
N3	541 (4.8%)	1716 (16%)	
Metastasis (M) stage, *N* (%)			< 0.001
M0	10,665 (95%)	5881 (54%)	
M1	575 (5.1%)	4958 (46%)	
Treatment			
Surgery, yes, *N* (%)	9276 (83%)	5732 (53%)	< 0.001
Chemotherapy, yes, *N* (%)	3740 (33%)	4329 (40%)	< 0.001
Radiotherapy, yes, *N* (%)	652 (5.8%)	905 (8.3%)	< 0.001
Multiple primary cancer, yes,* N *(%)	146 (1.3%)	378 (3.5%)	< 0.001
Family history of cancer, yes, *N* (%)	243 (2.2%)	215 (2.0%)	0.35
Lifestyle factors			
Smoking, *N* (%)			< 0.001
Never/former	10,422 (93%)	9519 (88%)	
Current	818 (7.3%)	1320 (12%)	
Alcohol, *N* (%)			< 0.001
Never/former	10,930 (97%)	10,280 (95%)	
Current	310 (2.8%)	559 (5.2%)	
Physical activity, minutes/week, median (IQR)^b^	90 (0–180)	15 (0–150)	< 0.001
Sleep duration, hours/day, median (IQR)^b^	7 (7–8)	7 (7–8)	< 0.001
Socioeconomic position			
Education, *N* (%)			< 0.001
Primary or below	2331 (21%)	3025 (28%)	
Secondary	6229 (55%)	5126 (47%)	
College or above	1549 (14%)	652 (6.0%)	
Not reported	1131 (10%)	2036 (19%)	
Employment, *N* (%)			< 0.001
Unemployed	6878 (61%)	8209 (76%)	
Employed	2327 (21%)	1187 (11%)	
Not reported	2035 (18%)	1443 (13%)	

*N* number, *SD* standard deviation

^a^Compared using Student’s *t*-tests

^b^Compared using Wilcoxon rank-sum tests

Table 2 shows that, after full adjustment, compared to cancer survivors diagnosed before the age of 50 years, those diagnosed at 50 years or older had a significantly higher risk of mortality (HR 1.71, 95% CI 1.61, 1.82; *P* < 0.001). No significant association of smoking with mortality risk was found. Compared with never/former alcohol use, current alcohol use was associated with a higher risk of all-cause mortality (HR 1.16, 95% CI 1.07, 1.27; *P* < 0.001). Both higher PA (HR 0.95 per 60 min, 95% CI 0.94, 0.96; *P* < 0.001) and longer sleep duration (HR 0.97 per 1 h, 95% CI 0.95, 0.99; *P* < 0.001) were associated with a lower all-cause mortality risk. Specifically, compared to cancer survivors sleeping 7 h/day, those who slept 8 h/day showed a lower all-cause mortality risk (HR 0.92, 95% CI 0.88, 0.96; *P* < 0.001).

**Table 2:** Associations of age at diagnosis and lifestyle factors with all-cause mortality risk

	*N*	Incidence rate/1000 person-years	Minimally adjusted HR (95% CI)^a^	Fully adjusted HR (95% CI)^b^	*P*^c^
Age at diagnosis, years					
< 50	5020	62.68	1.00	1.00	-
≥ 50	17,059	158.89	1.95 (1.84, 2.06)	1.71 (1.61, 1.82)	< 0.001
Smoking					
Never/former	19,941	128.46	1.00	1.00	-
Current	2138	187.96	1.00 (0.94, 1.06)	1.02 (0.96, 1.08)	0.57
Alcohol use					
Never/former	21,210	131.16	1.00	1.00	-
Current	869	203.35	1.14 (1.04, 1.24)	1.16 (1.07, 1.27)	< 0.001
Physical activity, minutes/week					
None (inactive)	9552	166.26	1.00	1.00	-
1–149 (insufficiently active)	5990	118.83	0.82 (0.78, 0.86)	0.84 (0.80, 0.88)	< 0.001
≥ 150 (active)	6537	105.73	0.73 (0.69, 0.76)	0.78 (0.74, 0.81)	< 0.001
Per 60 min increase			0.94 (0.93, 0.95)	0.95 (0.94, 0.96)	< 0.001
Sleep duration, hours/day					
≤ 5	633	197.64	1.19 (1.08, 1.32)	1.10 (0.99, 1.22)	0.07
6	2526	153.26	1.02 (0.96, 1.08)	0.96 (0.91, 1.02)	0.22
7	8670	139.81	1.00	1.00	-
8	9437	119.90	0.91 (0.88, 0.95)	0.92 (0.88, 0.96)	< 0.001
≥ 9	813	129.00	0.98 (0.88, 1.08)	0.95 (0.86, 1.05)	0.32
Per 1 h increase			0.95 (0.93, 0.97)	0.97 (0.95, 0.99)	< 0.001

*N* number, *HR* hazard ratio, *CI* confidence interval

^a^Adjusted for age, sex, and TNM stage

^b^Adjusted for age, sex, TNM stage, treatments, multiple primary cancer, family history of cancer, body mass index, education, and employment

^c^*P*-value was for the fully adjusted model

Similar associations with cancer-specific mortality risk were found (Supplementary Table 2). Additionally, cancer survivors sleeping ≤ 5 h/day showed a higher cancer-specific mortality risk than those sleeping 7 h/day (HR 1.15, 95% CI 1.03, 1.28; *P* = 0.01). Regarding CVD-specific mortality risk (Supplementary Table 3), PA was the only lifestyle factor significantly associated with mortality risk. Among survivors of digestive system cancers (Supplementary Table 4), in addition to diagnostic age, alcohol use, and PA, the shortest sleep duration (≤ 5 h/day) was associated with a higher risk of all-cause mortality, whereas no significant difference was observed between sleep durations of 7 and 8 h/day. For survivors of respiratory system cancers (Supplementary Table 5), only diagnostic age and PA showed significant associations with all-cause mortality risk.

Table 3 shows a significant multiplicative interaction between 1 SD increment in diagnostic age and 60 min increment in PA time (HR 0.98, 95% CI 0.97, 1.00; *P* = 0.008). Regarding additive interactions, a −0.05 (95% CI −0.07, −0.03) RERI of all-cause mortality was found due to the interaction, corresponding to a 3% (95% CI 2%, 5%) reduction in risk. Additionally, significant multiplicative interactions (HR 1.03, 95% CI 1.01, 1.05; *P* = 0.01) were observed between a 1-SD increment in diagnostic age and 1-h increase in sleep duration on all-cause mortality. However, no significant multiplicative or additive interactions were found between diagnostic age and smoking status, or between diagnostic age and alcohol use regarding all-cause mortality risk.

**Table 3:** Multiplicative and additive interactions between age at diagnosis and lifestyle factors on all-cause mortality risk

	Multiplicative interaction		Additive interaction		
	HR (95% CI)	*P*	RERI (95% CI)	AP (95% CI)	SI (95% CI)
Smoking (current)	0.96 (0.90, 1.03)	0.27	−0.02 (−0.14, 0.11)	−0.02 (−0.09, 0.06)	0.95 (0.72, 1.19)
Alcohol (current)	0.98 (0.89, 1.09)	0.76	0.05 (−0.16, 0.28)	0.03 (−0.10, 0.15)	1.08 (0.76, 1.42)
Physical activity, per 60 min/week	0.98 (0.97, 1.00)	0.008	−0.05 (−0.07, −0.03)	−0.03 (−0.05, −0.02)	0.89 (0.85, 0.93)
Sleep duration, per 1 h/day	1.03 (1.01, 1.05)	0.01	0.03 (−0.005, 0.05)	0.02 (−0.004, 0.05)	1.20 (−1.23, 4.18)

*HR* hazard ratio, *CI* confidence interval, *RERI* relative excess risk due to interaction, *AP* attributable proportion due to interaction, *SI* the synergy index

**Fig. 1:**
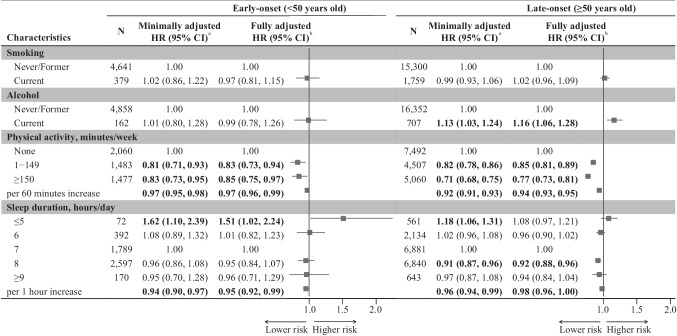
Associations of lifestyle factors with all-cause mortality risk in early-/late-onset cancer survivors. Abbreviations: N, number; HR, hazard ratio; CI, confidence interval. ^a^Adjusted for sex and TNM stage. ^b^Adjusted for sex, TNM stage, treatments, multiple primary cancer, family history of cancer, body mass index, education, and employment. Bold *P* < 0.05

As significant interactions between diagnostic age and lifestyle factors (PA time and sleep duration) were found, stratification analyses by early-/late-onset cancer survivors were performed. Figure 1 shows that a 60-min increase in PA time was associated with a 3% lower all-cause mortality risk in early-onset cancer survivors (95% CI 0.96, 0.99; *P* = 0.004) and 6% lower in late-onset cancer survivors (95% CI 0.93, 0.95; *P* < 0.001). Compared with inactivity, PA time of 150 min/week or more was associated with a 15% lower mortality risk (95% CI 0.75, 0.97; *P* = 0.02) in the early-onset group, and with a 23% lower risk (95% CI 0.73, 0.81; *P* < 0.001) in the late-onset group. Sleep duration showed a negative association with mortality risk in early-onset cancer survivors (HR 0.95, 95% CI 0.92, 0.99; *P* = 0.008). However, compared to a 7-h/day sleep duration, the lower all-cause mortality risk for those sleeping 8 h/day was observed only in the late-onset group (HR 0.92, 95% CI 0.88, 0.96; *P* < 0.001). In the early-onset group, the shortest sleep duration (≤ 5 h/day) was associated with a 51% higher risk (95% CI 1.02, 2.24; *P* = 0.04). Furthermore, smoking was not associated with all-cause mortality risk in either group, and the association between current alcohol use and higher all-cause mortality risk was only observed in the late-onset group.

After excluding participants who died within the first 2 months, consistent interaction effects with all-cause mortality (Supplementary Table 6) and associations in subgroups of early-/late-onset cancer survivors (Supplementary Table 7) were observed. Regarding cause-specific mortality, the shortest sleep duration was associated with a 59% higher risk of cancer-specific mortality in early-onset cancer survivors, compared to 14% higher in late-onset cancer survivors (Supplementary Table 2). PA was negatively associated with CVD-specific mortality risk only in late-onset cancer survivors (Supplementary Table 3). Stratification by cancer sites showed that the negative association between PA time and all-cause mortality risk was observed in both early-onset and late-onset cancer survivors with digestive system cancers, while the negative association between sleep duration and mortality risk was only observed in the late-onset group (Supplementary Table 8). The negative association between PA time and all-cause mortality risk was only observed in the late-onset cancer survivors with respiratory system cancers, but not in the early-onset group (Supplementary Table 9). Stratified analyses by metastasis status showed similar results (Supplementary Tables 10 and 11). Higher mortality risk among early-onset cancer survivors with the shortest sleep duration appeared only in those with metastasis, while the lower risk associated with 8-h sleep was seen only in late-onset cancer survivors without metastasis. Among survivors with low BMI (Supplementary Table 12), late-onset cancer survivors who had more PA time had a lower mortality risk, while early-onset survivors with the shortest sleep duration had a higher mortality risk. Among survivors with non-low BMI (Supplementary Table 13), late-onset survivors with more PA or 8-h sleep had a lower mortality risk.

**Fig. 2:**
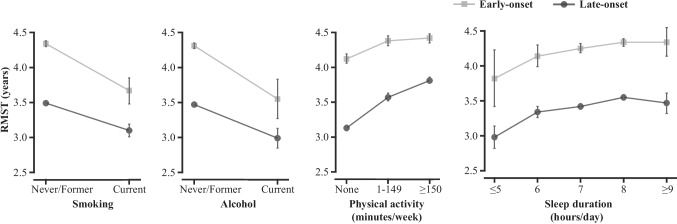
Five-year restricted mean survival time of early-/late-onset cancer survivors with different lifestyle factors. Abbreviation: RMST, restricted mean survival time

We estimated the 5-year RMST and RMST difference by early-/late-onset groups and lifestyle factors (Supplementary Table 14). The RMST over a 5-year follow-up was calculated as 4.29 years (95% CI 4.25, 4.33) for the early-onset cancer survivors compared to 3.45 years (95% CI 3.42, 3.48) for the late-onset group. As shown in Fig. 2, higher PA was significantly associated with a longer life expectancy. In the early-onset group, the RMST difference was 0.25 years (95% CI 0.16, 0.35) between insufficiently active and inactive survivors, and was 0.29 years (95% CI 0.20, 0.39) between active and inactive survivors. For the late-onset group, the RMST differences were more pronounced (insufficiently active vs. inactive: 0.45 years, 95% CI 0.38, 0.51; active vs. inactive: 0.69 years, 95% CI 0.62, 0.75). Early-onset cancer survivors sleeping ≤ 5 h/day had an estimated average survival time that was 0.43 years (95% CI 0.02, 0.84) shorter than those sleeping 7 h/day. In the late-onset survivors, those sleeping 8 h/day survived on average 0.13 years (95% CI 0.07, 0.19) longer than those sleeping 7 h/day.

## Discussion

In this city-wide cohort study, we identified an antagonistic effect between diagnostic age and PA time on all-cause mortality risk, complemented by a synergistic influence of diagnostic age and sleep duration. Compared to those with 7 h of sleep per day, early-onset cancer survivors with sleep of less than 5 h per day showed higher mortality risk, whereas late-onset cancer survivors with 8 h of sleep per day demonstrated a substantially lower risk. The findings highlight the roles of PA and sleep time interventions in reducing cancer mortality burden. Understanding the survival disparities attributable to lifestyle factors in early and late-onset cancer survivors is important for developing tailored health management strategies for this population.

The burden of late-onset cancer increases with the aging population [27], whereas the incidence of early-onset cancers, due to unhealthy lifestyles related to socioeconomic conditions, has increased dramatically under economic development [28, 29]. Premature morbidity and mortality in the early-onset group impose great societal and economic burdens [27]. Although early-onset cancer represents a unique spectrum of malignancy influenced by different biological and environmental factors [17, 30], studies on cancer mortality often aggregated them with late-onset cases, thereby obscuring interventions tailored specifically to early-/late-onset cancer survivors.

Our study extended the existing literature, explicitly showing a more pronounced negative association between PA and mortality risk. This association may be partly explained by exercise-induced attenuation of systemic inflammation and modulation of endocrine–metabolic pathways, including reductions in circulating inflammatory markers, insulin, and sex hormones, which suppress tumor-promoting signaling and improve cancer prognosis [31]. Chronic inflammation and metabolic dysregulation are age-related biological processes that may underlie the observed antagonistic interaction between diagnostic age and PA [32]. A cohort study of older cancer survivors (mean age = 65.1 years old) from the US National Health and Nutrition Examination Survey showed similar results, in that more PA was associated with a lower risk of all-cause mortality [8], which was also reported in another study of older cancer survivors with a mean age of 68.8 years [33], despite no significant interactions between two age groups (< or ≥ 71 years old) being found. The non-significant associations between PA and overall survival were also reported in cancer site-specific studies, i.e., in breast cancer survivors aged ≤ 58 years [34], or colorectal cancer survivors aged < 69 years [35]. One possible explanation is that older individuals are generally at a higher risk for CVD [36], while engaging in PA could have a more pronounced impact on reducing CVD risk [37]. Younger individuals have much lower CVD risk and might thus require a higher intensity or volume of PA, or a much larger sample size to observe similar benefits. Additionally, older cancer survivors are more likely to have comorbid conditions [38] and to be on various medications that could interact with both their CVD risk and the benefits derived from PA. The effects of PA on reducing CVD risk might be more evident in the context of these comorbidities.

Regarding sleep duration, our results were consistent with a cohort study of stage III colon cancer survivors, showing that those with sleeping less than 5 h per day had a higher mortality risk than those with 7 h per day [9]. However, another study reported no association between short sleep duration and survival in colorectal cancer survivors without adjusting for cancer stage [35]. One meta-analysis also showed a stronger association between short sleep duration and health outcomes in the general participants aged 65 years or younger [39]. Insufficient sleep can disturb circadian rhythm-regulated metabolic and endocrine pathways, leading to chronic inflammation and impaired immune surveillance, which contribute to tumor progression [40]. This effect appears to be more pronounced in early-onset cancer survivors, as circadian rhythm amplitude declines with age, and younger individuals exhibit stronger circadian oscillations and greater sensitivity to circadian disruption [41].

Furthermore, although declines in smoking prevalence have been followed by declines in smoking-related cancer death rates [42], no association between current smoking status and mortality risk was found in our study, probably due to the insufficient number of smokers in cancer survivors. Regarding the association between current alcohol use and higher mortality risk, a significant association was only observed in the late-onset cancer survivors. Late-onset cancer survivors may have had longer cumulative exposure to alcohol over their lifetime, potentially leading to more significant alcohol-related damage to their health [43].

The main strength of our study lies in the city-wide representative sample of Chinese cancer survivors, which allows the findings to be generalizable. Moreover, we used RMST to provide a direct and clinically interpretable measure of the average survival time within a specified time frame. RMST facilitates a clearer understanding for clinicians and patients of the potential impact of a risk factor on survival, compared to hazard ratios or survival probabilities at a single time point. In addition, RMST allows for straightforward comparison between groups by quantifying the absolute difference in mean survival time, aiding in the assessment of the impact of exposure variables. Nonetheless, some limitations should be acknowledged. First, all lifestyle information was self-reported using a simplified questionnaire with limited response categories, which may be subject to reporting bias. For example, PA in this study referred to total activity, as any intensity of PA provides health benefits according to the WHO Guidelines [44]. However, incorporating objective assessment tools and differentiating PA by intensity in future studies would help provide more specific exercise recommendations. Second, lifestyle factor assessment may not fully capture behavioral changes. In particular, changes in smoking and alcohol use before and after diagnosis would require longer follow-up to be accurately evaluated. Third, nutritional status, which reflects overall health condition and may influence survival outcomes [45], was not assessed, thus limiting further health guidance. Considering the flexibility of community-based practice, we attempted to explore the potential modifying effect of BMI. Further studies should incorporate more detailed and long-term assessments of lifestyle factors and overall health status to better understand their impact. Greater involvement of primary care in providing health education and behavioral interventions for cancer survivors is warranted.

## Conclusions

Our study showed that sufficient physical activity and sleep duration significantly contributed to the improved cancer prognosis, with the associations varying by age. The findings support the survival benefits of tailored interventions for early-onset and late-onset cancer survivors, highlighting the critical role of age-specific considerations in survivorship care.

## Supplementary Information

Below is the link to the electronic supplementary material.

ESM 1 Supplementary Material 1  (PDF 895 KB)

## Data Availability

Data supporting the study findings are not publicly available due to participant privacy protection but can be obtained from the Guangzhou Center for Disease Control and Prevention upon reasonable request.

## Abbreviations

PA: Physical activity
GCR: Guangzhou Cancer Registry
GZCDC: Guangzhou Center for Disease Control and Prevention
TNM: Tumor-node-metastasis
ICD-10: 10th Revision of the International Classification of Diseases
CVD: Cardiovascular disease
BMI: Body mass index
HR: Hazard ratio
CI: Confidence interval
RMST: Restricted mean survival time
SD: Standard deviation
RERI: Relative excess risk due to interaction
AP: Attributable proportion
SI: Synergy index
