# Supplementary material for: Age at diagnosis, lifestyle factors, and risk of mortality: a city-wide cohort study of cancer survivors
Source: Support Care Cancer. 2026 Jan 21;34(2):111. doi: 10.1007/s00520-026-10325-6 (PMC12819554; doi:10.1007/s00520-026-10325-6)
Supplement: Supplementary file 1 — Supplementary Material 1 (PDF 895 KB) [file 520_2026_10325_MOESM1_ESM.pdf]

Supplementary Materials

Supplementary Table 1. Distribution of cancer sites in the city-wide cohort (2010-2018) 2
Supplementary Table 2. Associations of lifestyle factors with cancer-specific mortality risk by early-/late-onset cancers 3
Supplementary Table 3. Associations of lifestyle factors with cardiovascular disease-specific mortality risk by early-/late-onset cancers 4
Supplementary Table 4. Associations of age at diagnosis and lifestyle factors with all-cause mortality risk in digestive system cancer survivors 5
Supplementary Table 5. Associations of age at diagnosis and lifestyle factors with all-cause mortality risk in respiratory system cancer survivors 6
Supplementary Table 6. Multiplicative and additive interactions between age at diagnosis and lifestyle factors on all-cause mortality risk excluding deaths within the first 2 months 7
Supplementary Table 7. Associations of lifestyle factors with all-cause mortality risk excluding deaths within the first 2 months 8
Supplementary Table 8. Associations of lifestyle factors with all-cause mortality risk in early-/late-onset digestive system cancer survivors 9
Supplementary Table 9. Associations of lifestyle factors with all-cause mortality risk in early-/late-onset respiratory system cancer survivors 10
Supplementary Table 10. Associations of lifestyle factors with all-cause mortality risk in early-/late-onset cancer survivors with metastasis 11
Supplementary Table 11. Associations of lifestyle factors with all-cause mortality risk in early-/late-onset cancer survivors without metastasis 12
Supplementary Table 12. Associations of lifestyle factors with all-cause mortality risk in early-/late-onset cancer survivors with low body mass index 13
Supplementary Table 13. Associations of lifestyle factors with all-cause mortality risk in early-/late-onset cancer survivors with non-low body mass index 14
Supplementary Table 14. Measures of 5-year survival outcomes using Kaplan-Meier estimator for the univariate association 15

Supplementary Table 1. Distribution of cancer sites in the city-wide cohort (2010-2018)

	Frequency, N (%)		
**Cancer sites**	**All cancers**	**Early-onset cancers**	**Late-onset cancers**
Oral cavity & pharynx	1,860 (8.42)	1,261 (12.14)	599 (5.12)
Digestive system	8,498 (38.49)	2,986 (28.74)	5,512 (47.16)
Respiratory system	4,238 (19.19)	1,461 (14.06)	2,777 (23.76)
Bones & joints	36 (0.16)	24 (0.23)	12 (0.10)
Soft tissue	75 (0.34)	40 (0.38)	35 (0.30)
Skin	168 (0.76)	37 (0.36)	131 (1.12)
Breast	3,822 (17.31)	2,709 (26.07)	1,113 (9.52)
Genital system	1,447 (6.55)	682 (6.56)	765 (6.54)
Urinary system	557 (2.52)	176 (1.69)	381 (3.26)
Eye & orbit	2 (0.01)	2 (0.02)	0 (0.00)
Brain & other nervous system	48 (0.22)	26 (0.25)	22 (0.19)
Endocrine system	943 (4.27)	824 (7.93)	119 (1.02)
Lymphoma	126 (0.57)	54 (0.52)	72 (0.62)
Myeloma	23 (0.10)	4 (0.04)	19 (0.16)
Leukemia	37 (0.17)	23 (0.22)	14 (0.12)
Other & unspecified primary sites	199 (0.90)	81 (0.78)	118 (1.01)
**Total**	22,079 (100.00)	10,390 (100.00)	11,689 (100.00)

Supplementary Table 2. Associations of lifestyle factors with cancer-specific mortality risk by early-/late-onset cancers

	All			Early-onset (<50 years old)			Late-onset (≥50 years old)		
	Minimally adjusted HR (95% CI) [a]	Fully adjusted HR (95% CI) [b]	*P* [c]	Minimally adjusted HR (95% CI) [a]	Fully adjusted HR (95% CI) [b]	*P* [c]	Minimally adjusted HR (95% CI) [a]	Fully adjusted HR (95% CI) [b]	*P* [c]
**Smoking**									
Never/Former	1.00	1.00	-	1.00	1.00	-	1.00	1.00	-
Current	1.02 (0.95, 1.09)	1.02 (0.96, 1.09)	0.46	0.99 (0.82, 1.18)	0.94 (0.78, 1.13)	0.48	1.02 (0.95, 1.09)	1.04 (0.97, 1.12)	0.23
**Alcohol**									
Never/Former	1.00	1.00	-	1.00	1.00	-	1.00	1.00	-
Current	1.16 (1.05, 1.27)	1.18 (1.07, 1.29)	<0.001	0.95 (0.74, 1.21)	0.92 (0.72, 1.19)	0.54	1.18 (1.06, 1.30)	1.21 (1.09, 1.34)	<0.001
**Physical activity, minutes/week**									
None (inactive)	1.00	1.00	-	1.00	1.00	-	1.00	1.00	-
1-149 (insufficiently active)	0.82 (0.78, 0.87)	0.86 (0.82, 0.90)	<0.001	0.81 (0.71, 0.93)	0.82 (0.72, 0.94)	0.005	0.83 (0.78, 0.87)	0.86 (0.81, 0.91)	<0.001
≥150 (active)	0.72 (0.69, 0.76)	0.78 (0.74, 0.82)	<0.001	0.82 (0.72, 0.94)	0.85 (0.74, 0.97)	0.02	0.71 (0.67, 0.75)	0.76 (0.72, 0.80)	<0.001
per 60 minutes increase	0.94 (0.93, 0.95)	0.95 (0.94, 0.96)	<0.001	0.95 (0.92, 0.98)	0.96 (0.93, 0.99)	0.009	0.93 (0.92, 0.95)	0.95 (0.94, 0.96)	<0.001
**Sleep duration, hours/day**									
≤5	1.25 (1.12, 1.40)	1.15 (1.03, 1.28)	0.01	1.69 (1.15, 2.50)	1.59 (1.08, 2.36)	0.02	1.23 (1.10, 1.38)	1.14 (1.02, 1.28)	0.03
6	1.01 (0.95, 1.08)	0.95 (0.89, 1.02)	0.16	1.11 (0.91, 1.36)	1.03 (0.84, 1.27)	0.78	1.01 (0.94, 1.08)	0.95 (0.89, 1.02)	0.19
7	1.00	1.00	-	1.00	1.00	-	1.00	1.00	-
8	0.92 (0.88, 0.97)	0.94 (0.90, 0.98)	0.007	0.97 (0.86, 1.10)	0.96 (0.85, 1.08)	0.51	0.92 (0.87, 0.97)	0.93 (0.89, 0.98)	0.006
≥9	0.97 (0.87, 1.09)	0.94 (0.84, 1.05)	0.27	0.96 (0.71, 1.31)	0.97 (0.71, 1.32)	0.83	0.97 (0.86, 1.09)	0.94 (0.83, 1.06)	0.30
per 1 hour increase	0.95 (0.93, 0.97)	0.97 (0.95, 0.99)	0.004	0.92 (0.86, 0.98)	0.93 (0.87, 0.99)	0.03	0.96 (0.93, 0.97)	0.97 (0.95, 0.99)	0.007

Abbreviations: N, number; HR, hazard ratio; CI, confidence interval.

[a] Adjusted for age (not in the early-/late-onset subgroups), sex and TNM stage.

[b] Adjusted for age (not in the early-/late-onset subgroups), sex, TNM stage, treatments, multiple primary cancer, family history of cancer, body mass index, education, and employment.

[c] *P*-value was for the fully adjusted model.

Supplementary Table 3. Associations of lifestyle factors with cardiovascular disease-specific mortality risk by early-/late-onset cancers

	All			Early-onset (<50 years old)			Late-onset (≥50 years old)		
	Minimally adjusted HR (95% CI) [a]	Fully adjusted HR (95% CI) [b]	*P* [c]	Minimally adjusted HR (95% CI) [a]	Fully adjusted HR (95% CI) [b]	*P* [c]	Minimally adjusted HR (95% CI) [a]	Fully adjusted HR (95% CI) [b]	*P* [c]
**Smoking**									
Never/Former	1.00	1.00	-	1.00	1.00	-	1.00	1.00	-
Current	0.98 (0.75, 1.29)	1.00 (0.76, 1.31)	0.99	0.77 (0.14, 4.23)	0.79 (0.13, 4.75)	0.79	0.98 (0.74, 1.29)	0.99 (0.75, 1.31)	0.95
**Alcohol**									
Never/Former	1.00	1.00	-	1.00	1.00	-	1.00	1.00	-
Current	0.73 (0.45, 1.17)	0.75 (0.47, 1.20)	0.23	0.00 (0.00, Inf)	0.00 (0.00, Inf)	>0.99	0.75 (0.47, 1.20)	0.77 (0.48, 1.23)	0.28
**Physical activity, minutes/week**									
None (inactive)	1.00	1.00	-	1.00	1.00	-	1.00	1.00	-
1-149 (insufficiently active)	0.73 (0.60, 0.88)	0.77 (0.63, 0.94)	0.01	0.66 (0.17, 2.60)	0.52 (0.12, 2.17)	0.37	0.73 (0.60, 0.89)	0.78 (0.64, 0.95)	0.01
≥150 (active)	0.59 (0.48, 0.72)	0.66 (0.54, 0.81)	<0.001	1.11 (0.31, 3.94)	1.11 (0.31, 3.98)	0.88	0.59 (0.48, 0.72)	0.65 (0.53, 0.81)	<0.001
per 60 minutes increase	0.87 (0.83, 0.91)	0.89 (0.85, 0.94)	<0.001	1.00 (0.75, 1.34)	1.00 (0.73, 1.36)	0.97	0.87 (0.83, 0.91)	0.89 (0.85, 0.94)	<0.001
**Sleep duration, hours/day**									
≤5	0.98 (0.61, 1.59)	0.83 (0.51, 1.34)	0.44	0.00 (0.00, Inf)	0.00 (0.00, Inf)	>0.99	1.00 (0.62, 1.62)	0.84 (0.52, 1.36)	0.49
6	1.06 (0.82, 1.36)	0.94 (0.73, 1.21)	0.64	0.41 (0.05, 3.62)	0.50 (0.06, 4.51)	0.54	1.07 (0.83, 1.38)	0.95 (0.74, 1.22)	0.69
7	1.00	1.00	-	1.00	1.00	-	1.00	1.00	-
8	0.87 (0.72, 1.04)	0.86 (0.72, 1.04)	0.12	0.57 (0.18, 1.83)	0.64 (0.20, 2.09)	0.46	0.88 (0.73, 1.06)	0.87 (0.72, 1.05)	0.15
≥9	1.37 (0.96, 1.94)	1.25 (0.88, 1.78)	0.21	0.72 (0.07, 7.13)	0.76 (0.06, 9.70)	0.83	1.37 (0.96, 1.96)	1.25 (0.87, 1.78)	0.23
per 1 hour increase	1.00 (0.92, 1.09)	1.02 (0.94, 1.11)	0.59	0.99 (0.54, 1.81)	1.00 (0.53, 1.88)	0.99	1.00 (0.92, 1.09)	1.02 (0.94, 1.11)	0.59

Abbreviations: N, number; HR, hazard ratio; CI, confidence interval.

[a] Adjusted for age (not in the early-/late-onset subgroups), sex and TNM stage.

[b] Adjusted for age (not in the early-/late-onset subgroups), sex, TNM stage, treatments, multiple primary cancer, family history of cancer, body mass index, education, and employment.

[c] *P*-value was for the fully adjusted model.

Supplementary Table 4. Associations of age at diagnosis and lifestyle factors with all-cause mortality risk in digestive system cancer survivors

	N	Incidence rate /1000 person-years	Minimally adjusted HR (95% CI) [a]	Fully adjusted HR (95% CI) [b]	*P* [c]
**Age at diagnosis, years**					
<50	1,149	122.73	1.00	1.00	-
≥50	7,349	162.30	1.39 (1.27, 1.53)	1.25 (1.13, 1.38)	<0.001
**Smoking**					
Never/Former	7,535	154.78	1.00	1.00	-
Current	963	173.36	1.02 (0.93, 1.12)	1.02 (0.93, 1.11)	0.75
**Alcohol**					
Never/Former	8,100	154.88	1.00	1.00	-
Current	398	200.64	1.17 (1.03, 1.34)	1.18 (1.04, 1.35)	0.01
**Physical activity, minutes/week**					
None (inactive)	3,730	193.70	1.00	1.00	-
1-149 (insufficiently active)	2,219	144.03	0.79 (0.73, 0.84)	0.82 (0.76, 0.88)	<0.001
≥150 (active)	2,549	122.01	0.69 (0.65, 0.74)	0.75 (0.70, 0.81)	<0.001
per 60 minutes increase			0.93 (0.91, 0.94)	0.94 (0.93, 0.96)	<0.001
**Sleep duration, hours/day**					
≤5	250	227.04	1.34 (1.14, 1.57)	1.27 (1.08, 1.49)	0.003
6	995	166.56	1.03 (0.94, 1.13)	0.97 (0.89, 1.07)	0.58
7	3,453	157.76	1.00	1.00	-
8	3,493	148.70	0.93 (0.87, 0.99)	0.96 (0.90, 1.02)	0.19
≥9	307	159.72	0.95 (0.82, 1.11)	0.95 (0.81, 1.11)	0.51
per 1 hour increase			0.94 (0.91, 0.97)	0.97 (0.94, 1.00)	0.03

Abbreviations: N, number; HR, hazard ratio; CI, confidence interval.

[a] Adjusted for age, sex and TNM stage.

[b] Adjusted for age, sex, TNM stage, treatments, multiple primary cancer, family history of cancer, body mass index, education, and employment.

[c] *P*-value was for the fully adjusted model.

Supplementary Table 5. Associations of age at diagnosis and lifestyle factors with all-cause mortality risk in respiratory system cancer survivors

	N	Incidence rate /1000 person-years	Minimally adjusted HR (95% CI) [a]	Fully adjusted HR (95% CI) [b]	*P* [c]
**Age at diagnosis, years**					
<50	438	219.5	1.00	1.00	-
≥50	3,800	309.74	1.42 (1.26, 1.60)	1.22 (1.07, 1.38)	0.002
**Smoking**					
Never/Former	3,644	291.89	1.00	1.00	-
Current	594	348.27	1.04 (0.94, 1.15)	1.06 (0.96, 1.17)	0.25
**Alcohol**					
Never/Former	3,983	297.1	1.00	1.00	-
Current	255	330.13	1.05 (0.91, 1.21)	1.11 (0.96, 1.28)	0.16
**Physical activity, minutes/week**					
None (inactive)	2,005	361.98	1.00	1.00	-
1-149 (insufficiently active)	1,118	265.52	0.84 (0.77, 0.91)	0.88 (0.81, 0.95)	0.002
≥150 (active)	1,115	243.28	0.76 (0.70, 0.82)	0.82 (0.75, 0.89)	<0.001
per 60 minutes increase			0.95 (0.93, 0.97)	0.97 (0.95, 0.98)	<0.001
**Sleep duration, hours/day**					
≤5	153	375.61	1.14 (0.95, 1.36)	1.05 (0.88, 1.25)	0.59
6	515	318.79	1.04 (0.94, 1.17)	0.97 (0.87, 1.08)	0.56
7	1,724	309.07	1.00	1.00	-
8	1,700	279.17	0.93 (0.86, 1.01)	0.95 (0.88, 1.03)	0.21
≥9	146	280.54	1.00 (0.83, 1.21)	0.98 (0.81, 1.19)	0.84
per 1 hour increase			0.96 (0.93, 1.00)	0.98 (0.95, 1.02)	0.37

Abbreviations: N, number; HR, hazard ratio; CI, confidence interval.

[a] Adjusted for age, sex and TNM stage.

[b] Adjusted for age, sex, TNM stage, treatments, multiple primary cancer, family history of cancer, body mass index, education, and employment.

[c] *P*-value was for the fully adjusted model.

Supplementary Table 6. Multiplicative and additive interactions between age at diagnosis and lifestyle factors on all-cause mortality risk excluding deaths within the first 2 months

	Multiplicative interaction		Additive interaction		
	**HR (95% CI)**	***P***	**RERI (95% CI)**	**AP (95% CI)**	**SI (95% CI)**
**Smoking** (current)	0.95 (0.89, 1.02)	0.14	-0.04 (-0.16, 0.08)	-0.03 (-0.11, 0.05)	0.92 (0.71, 1.15)
**Alcohol** (current)	0.98 (0.89, 1.09)	0.71	0.05 (-0.15, 0.30)	0.03 (-0.11, 0.14)	1.07 (0.77, 1.42)
**Physical activity**, per 60 minutes/week	0.99 (0.97, 1.00)	0.01	-0.05 (-0.07, -0.03)	-0.03 (-0.05, -0.02)	0.90 (0.86, 0.94)
**Sleep duration**, per 1 hour/day	1.03 (1.00, 1.05)	0.02	0.03 (-0.005, 0.05)	0.02 (-0.001, 0.05)	1.18 (-1.22, 3.28)

Abbreviations: HR, hazard ratio; CI, confidence interval; RERI, relative excess risk due to interaction; AP, attributable proportion due to interaction; SI, the synergy index.

Supplementary Table 7. Associations of lifestyle factors with all-cause mortality risk excluding deaths within the first 2 months

	All			Early-onset (<50 years old)			Late-onset (≥50 years old)		
	Minimally adjusted HR (95% CI) [a]	Fully adjusted HR (95% CI) [b]	*P* [c]	Minimally adjusted HR (95% CI) [a]	Fully adjusted HR (95% CI) [b]	*P* [c]	Minimally adjusted HR (95% CI) [a]	Fully adjusted HR (95% CI) [b]	*P* [c]
**Smoking**									
Never/Former	1.00	1.00	-	1.00	1.00	-	1.00	1.00	-
Current	1.00 (0.94, 1.06)	1.02 (0.96, 1.08)	0.58	1.02 (0.86, 1.21)	0.97 (0.81, 1.15)	0.71	0.99 (0.93, 1.06)	1.02 (0.95, 1.09)	0.57
**Alcohol**									
Never/Former	1.00	1.00	-	1.00	1.00	-	1.00	1.00	-
Current	1.14 (1.05, 1.25)	1.17 (1.07, 1.28)	<0.001	1.01 (0.79, 1.28)	0.99 (0.78, 1.26)	0.95	1.14 (1.04, 1.25)	1.17 (1.07, 1.29)	0.001
**Physical activity, minutes/week**									
None (inactive)	1.00	1.00	-	1.00	1.00	-	1.00	1.00	-
1-149 (insufficiently active)	0.82 (0.79, 0.86)	0.85 (0.81, 0.89)	<0.001	0.82 (0.72, 0.93)	0.83 (0.73, 0.95)	0.005	0.82 (0.78, 0.87)	0.85 (0.81, 0.90)	<0.001
⩾150 (active)	0.74 (0.70, 0.77)	0.79 (0.75, 0.82)	<0.001	0.84 (0.73, 0.95)	0.86 (0.75, 0.98)	0.03	0.72 (0.69, 0.76)	0.78 (0.74, 0.82)	<0.001
per 60 minutes increase	0.94 (0.93, 0.95)	0.95 (0.94, 0.96)	<0.001	0.96 (0.93, 0.99)	0.97 (0.94, 1.00)	0.03	0.94 (0.93, 0.95)	0.95 (0.94, 0.96)	<0.001
**Sleep duration, hours/day**									
⩽5	1.19 (1.07, 1.32)	1.10 (0.99, 1.22)	0.07	1.64 (1.11, 2.42)	1.54 (1.04, 2.27)	0.03	1.17 (1.05, 1.31)	1.08 (0.97, 1.21)	0.16
6	1.02 (0.96, 1.08)	0.96 (0.91, 1.03)	0.24	1.08 (0.89, 1.32)	1.01 (0.83, 1.24)	0.91	1.02 (0.96, 1.09)	0.96 (0.90, 1.02)	0.22
7	1.00	1.00	-	1.00	1.00	-	1.00	1.00	-
8	0.92 (0.88, 0.96)	0.92 (0.88, 0.96)	<0.001	0.96 (0.85, 1.08)	0.95 (0.84, 1.06)	0.35	0.92 (0.88, 0.96)	0.92 (0.88, 0.97)	<0.001
⩾9	0.97 (0.88, 1.08)	0.95 (0.85, 1.05)	0.29	0.95 (0.71, 1.29)	0.96 (0.71, 1.30)	0.81	0.96 (0.86, 1.07)	0.93 (0.84, 1.04)	0.21
per 1 hour increase	0.95 (0.93, 0.97)	0.97 (0.95, 0.99)	0.001	0.92 (0.87, 0.98)	0.93 (0.87, 0.99)	0.03	0.95 (0.94, 0.97)	0.97 (0.95, 0.99)	0.01

Abbreviations: N, number; HR, hazard ratio; CI, confidence interval.

[a] Adjusted for age (not in the early-/late-onset subgroups), sex and TNM stage.

[b] Adjusted for age (not in the early-/late-onset subgroups), sex, TNM stage, treatments, multiple primary cancer, family history of cancer, body mass index, education, and employment.

[c] *P*-value was for the fully adjusted model.

Supplementary Table 8. Associations of lifestyle factors with all-cause mortality risk in early-/late-onset digestive system cancer survivors

	Early-onset (<50 years old)				Late-onset (≥50 years old)			
	N	Minimally adjusted HR (95% CI) [a]	Fully adjusted HR (95% CI) [b]	*P* [c]	N	Minimally adjusted HR (95% CI) [a]	Fully adjusted HR (95% CI) [b]	*P* [c]
**Smoking**								
Never/Former	1,010	1.00	1.00	-	6,525	1.00	1.00	-
Current	139	1.61 (1.25, 2.08)	1.49 (1.15, 1.93)	0.003	824	0.95 (0.86, 1.05)	0.96 (0.87, 1.06)	0.45
**Alcohol**								
Never/Former	1,084	1.00	1.00	-	7,016	1.00	1.00	-
Current	65	1.40 (0.99, 1.97)	1.30 (0.91, 1.85)	0.14	333	1.15 (1.00, 1.32)	1.15 (1.00, 1.33)	0.045
**Physical activity, minutes/week**								
None (inactive)	480	1.00	1.00	-	3,250	1.00	1.00	-
1-149 (insufficiently active)	335	0.77 (0.62, 0.96)	0.83 (0.67, 1.03)	0.09	1,884	0.80 (0.74, 0.86)	0.82 (0.76, 0.89)	<0.001
≥150 (active)	334	0.74 (0.60, 0.92)	0.80 (0.64, 1.00)	0.05	2,215	0.69 (0.64, 0.74)	0.75 (0.70, 0.81)	<0.001
per 60 minutes increase		0.94 (0.89, 0.99)	0.95 (0.91, 1.00)	0.04		0.92 (0.91, 0.94)	0.94 (0.93, 0.96)	<0.001
**Sleep duration, hours/day**								
≤5	12	1.50 (0.73, 3.06)	1.52 (0.73, 3.13)	0.26	238	1.29 (1.10, 1.53)	1.34 (1.12, 1.60)	0.001
6	79	0.82 (0.56, 1.19)	0.77 (0.52, 1.13)	0.18	916	1.04 (0.95, 1.15)	1.02 (0.91, 1.13)	0.78
7	424	1.00	1.00	-	3,029	1.00	1.00	-
8	590	0.95 (0.79, 1.16)	0.95 (0.79, 1.16)	0.63	2,903	0.94 (0.88, 1.01)	0.97 (0.89, 1.04)	0.37
≥9	44	1.05 (0.67, 1.65)	1.11 (0.71, 1.75)	0.64	263	0.96 (0.81, 1.13)	0.94 (0.78, 1.14)	0.55
per 1 hour increase		0.99 (0.88, 1.11)	1.00 (0.99, 1.13)	0.95		0.95 (0.92, 0.98)	0.97 (0.94, 1.00)	0.03

Abbreviations: N, number; HR, hazard ratio; CI, confidence interval.

[a] Adjusted for sex and TNM stage.

[b] Adjusted for sex, TNM stage, treatments, multiple primary cancer, family history of cancer, body mass index, education, and employment.

[c] *P*-value was for the fully adjusted model.

Supplementary Table 9. Associations of lifestyle factors with all-cause mortality risk in early-/late-onset respiratory system cancer survivors

	Early-onset (<50 years old)				Late-onset (≥50 years old)			
	N	Minimally adjusted HR (95% CI) [a]	Fully adjusted HR (95% CI) [b]	*P* [c]	N	Minimally adjusted HR (95% CI) [a]	Fully adjusted HR (95% CI) [b]	*P* [c]
**Smoking**								
Never/Former	387	1.00	1.00	-	3,257	1.00	1.00	-
Current	51	1.02 (0.70, 1.50)	0.97 (0.65, 1.43)	0.87	543	1.04 (0.94, 1.16)	1.07 (0.96, 1.19)	0.21
**Alcohol**								
Never/Former	415	1.00	1.00	-	3,568	1.00	1.00	-
Current	23	0.78 (0.46, 1.30)	0.72 (0.42, 1.24)	0.24	232	1.08 (0.93, 1.26)	1.16 (0.99, 1.34)	0.06
**Physical activity, minutes/week**								
None (inactive)	199	1.00	1.00	-	1,806	1.00	1.00	-
1-149 (insufficiently active)	123	0.81 (0.61, 1.08)	0.82 (0.61, 1.11)	0.21	995	0.84 (0.77, 0.92)	0.88 (0.81, 0.96)	0.004
≥150 (active)	116	0.81 (0.61, 1.07)	0.79 (0.60, 1.06)	0.12	999	0.75 (0.69, 0.82)	0.81 (0.74, 0.89)	<0.001
per 60 minutes increase		0.97 (0.91, 1.04)	0.97 (0.91, 1.03)	0.29		0.95 (0.93, 0.97)	0.96 (0.95, 0.98)	<0.001
**Sleep duration, hours/day**								
≤5	8	1.45 (0.65, 3.20)	1.18 (0.52, 2.67)	0.69	145	1.10 (0.92, 1.32)	1.06 (0.88, 1.29)	0.52
6	44	1.23 (0.82, 1.85)	1.12 (0.74, 1.71)	0.59	471	1.03 (0.92, 1.15)	0.96 (0.85, 1.08)	0.47
7	162	1.00	1.00	-	1,562	1.00	1.00	-
8	213	0.98 (0.76, 1.26)	0.92 (0.71, 1.19)	0.53	1,487	0.94 (0.87, 1.02)	0.97 (0.89, 1.05)	0.41
≥9	11	1.42 (0.71, 2.85)	1.18 (0.55, 2.51)	0.67	135	0.98 (0.80, 1.19)	0.97 (0.78, 1.20)	0.77
per 1 hour increase		0.95 (0.82, 1.08)	0.94 (0.82, 1.08)	0.41		0.97 (0.94, 1.00)	0.99 (0.96, 1.02)	0.55

Abbreviations: N, number; HR, hazard ratio; CI, confidence interval.

[a] Adjusted for sex and TNM stage.

[b] Adjusted for sex, TNM stage, treatments, multiple primary cancer, family history of cancer, body mass index, education, and employment.

[c] *P*-value was for the fully adjusted model.

Supplementary Table 10. Associations of lifestyle factors with all-cause mortality risk in early-/late-onset cancer survivors with metastasis

	Early-onset (<50 years old)				Late-onset (≥50 years old)			
	N	Minimally adjusted HR (95% CI) [a]	Fully adjusted HR (95% CI) [b]	*P* [c]	N	Minimally adjusted HR (95% CI) [a]	Fully adjusted HR (95% CI) [b]	*P* [c]
**Smoking**								
Never/Former	704	1.00	1.00	-	4,192	1.00	1.00	-
Current	84	1.04 (0.80, 1.34)	0.93 (0.71, 1.21)	0.58	553	0.96 (0.87, 1.06)	0.98 (0.89, 1.08)	0.66
**Alcohol**								
Never/Former	746	1.00	1.00	-	4,523	1.00	1.00	-
Current	42	0.92 (0.65, 1.30)	0.87 (0.61, 1.24)	0.45	222	1.15 (1.00, 1.33)	1.19 (1.03, 1.37)	0.02
**Physical activity, minutes/week**								
None (inactive)	388	1.00	1.00	-	2,436	1.00	1.00	-
1-149 (insufficiently active)	203	0.76 (0.62, 0.91)	0.78 (0.64, 0.95)	0.01	1,164	0.89 (0.82, 0.95)	0.91 (0.85, 0.98)	0.01
≥150 (active)	197	0.82 (0.68, 0.99)	0.83 (0.69, 1.01)	0.06	1,145	0.74 (0.69, 0.80)	0.77 (0.72, 0.83)	<0.001
per 60 minutes increase		0.95 (0.90, 0.99)	0.95 (0.91, 0.99)	0.02		0.94 (0.92, 0.96)	0.95 (0.93, 0.96)	<0.001
**Sleep duration, hours/day**								
≤5	20	1.74 (1.08, 2.78)	1.80 (1.12, 2.91)	0.02	192	1.10 (0.94, 1.28)	1.06 (0.91, 1.25)	0.45
6	77	1.22 (0.93, 1.61)	1.17 (0.88, 1.55)	0.28	602	0.94 (0.86, 1.04)	0.90 (0.81, 0.99)	0.03
7	301	1.00	1.00	-	1,934	1.00	1.00	-
8	371	1.07 (0.90, 1.27)	1.06 (0.89, 1.26)	0.50	1,845	0.93 (0.87, 0.99)	0.95 (0.88, 1.02)	0.14
≥9	19	0.89 (0.53, 1.49)	0.84 (0.50, 1.42)	0.52	172	1.08 (0.92, 1.27)	1.07 (0.91, 1.27)	0.42
per 1 hour increase		0.92 (0.85, 1.01)	0.92 (0.84, 1.00)	0.05		0.99 (0.96, 1.02)	1.00 (0.97, 1.03)	0.82

Abbreviations: N, number; HR, hazard ratio; CI, confidence interval.

[a] Adjusted for sex and TNM stage.

[b] Adjusted for sex, TNM stage, treatments, multiple primary cancer, family history of cancer, body mass index, education, and employment.

[c] *P*-value was for the fully adjusted model.

Supplementary Table 11. Associations of lifestyle factors with all-cause mortality risk in early-/late-onset cancer survivors without metastasis

	Early-onset (<50 years old)				Late-onset (≥50 years old)			
	N	Minimally adjusted HR (95% CI) [a]	Fully adjusted HR (95% CI) [b]	*P* [c]	N	Minimally adjusted HR (95% CI) [a]	Fully adjusted HR (95% CI) [b]	*P* [c]
**Smoking**								
Never/Former	3,937	1.00	1.00	-	11,108	1.00	1.00	-
Current	295	1.04 (0.82, 1.32)	1.07 (0.84, 1.37)	0.56	1,206	1.01 (0.93, 1.10)	1.03 (0.94, 1.12)	0.51
**Alcohol**								
Never/Former	4,112	1.00	1.00	-	11,829	1.00	1.00	-
Current	120	1.33 (0.96, 1.83)	1.36 (0.98, 1.88)	0.07	485	1.11 (0.98, 1.25)	1.09 (0.97, 1.24)	0.16
**Physical activity, minutes/week**								
None (inactive)	1,672	1.00	1.00	-	5,056	1.00	1.00	-
1-149 (insufficiently active)	1,280	0.82 (0.68, 0.98)	0.81 (0.68, 0.97)	0.02	3,343	0.77 (0.72, 0.83)	0.80 (0.75, 0.86)	<0.001
≥150 (active)	1,280	0.85 (0.71, 1.02)	0.85 (0.71, 1.03)	0.10	3,915	0.70 (0.66, 0.75)	0.77 (0.72, 0.82)	<0.001
per 60 minutes increase		0.97 (0.93, 1.01)	0.97 (0.93, 1.01)	0.21		0.94 (0.92, 0.95)	0.95 (0.94, 0.97)	<0.001
**Sleep duration, hours/day**								
≤5	52	1.21 (0.60, 2.44)	1.00 (0.49, 2.03)	0.99	369	1.21 (1.04, 1.40)	1.17 (0.99, 1.39)	0.06
6	315	0.87 (0.66, 1.17)	0.86 (0.64, 1.15)	0.30	1,532	1.09 (1.00, 1.18)	1.04 (0.94, 1.15)	0.44
7	1,488	1.00	1.00	-	4,947	1.00	1.00	-
8	2,226	0.84 (0.71, 0.99)	0.84 (0.72, 1.00)	0.05	4,995	0.90 (0.85, 0.96)	0.91 (0.85, 0.98)	0.008
≥9	151	0.97 (0.67, 1.41)	1.05 (0.72, 1.54)	0.78	471	0.91 (0.79, 1.06)	0.85 (0.71, 1.02)	0.08
per 1 hour increase		0.94 (0.86, 1.03)	0.96 (0.88, 1.05)	0.41		0.93 (0.90, 0.96)	0.95 (0.92, 0.98)	<0.001

Abbreviations: N, number; HR, hazard ratio; CI, confidence interval.

[a] Adjusted for sex and TNM stage.

[b] Adjusted for sex, TNM stage, treatments, multiple primary cancer, family history of cancer, body mass index, education, and employment.

[c] *P*-value was for the fully adjusted model.

Supplementary Table 12. Associations of lifestyle factors with all-cause mortality risk in early-/late-onset cancer survivors with low body mass index

	Early-onset (<50 years old)				Late-onset (≥50 years old)			
	N	Minimally adjusted HR (95% CI) [a]	Fully adjusted HR (95% CI) [b]	*P* [c]	N	Minimally adjusted HR (95% CI) [a]	Fully adjusted HR (95% CI) [b]	*P* [c]
**Smoking**								
Never/Former	368	1.00	1.00	-	2,236	1.00	1.00	-
Current	28	1.11 (0.58, 2.12)	0.95 (0.48, 1.91)	0.90	221	0.98 (0.83, 1.15)	1.05 (0.89, 1.24)	0.54
**Alcohol**								
Never/Former	385	1.00	1.00	-	2,388	1.00	1.00	-
Current	11	2.67 (1.14, 6.29)	2.75 (1.15, 6.59)	0.02	69	1.31 (1.00, 1.70)	1.35 (1.04, 1.76)	0.02
**Physical activity, minutes/week**								
None (inactive)	165	1.00	1.00	-	1,270	1.00	1.00	-
1-149 (insufficiently active)	123	0.82 (0.56, 1.20)	0.95 (0.63, 1.42)	0.80	612	0.77 (0.69, 0.87)	0.83 (0.74, 0.93)	0.001
≥150 (active)	108	0.63 (0.40, 0.99)	0.64 (0.40, 1.04)	0.07	575	0.69 (0.61, 0.78)	0.75 (0.67, 0.85)	<0.001
per 60 minutes increase		0.92 (0.82, 1.02)	0.92 (0.82, 1.03)	0.15		0.92 (0.89, 0.94)	0.94 (0.91, 0.96)	<0.001
**Sleep duration, hours/day**								
≤5	7	3.79 (1.57, 9.16)	6.89 (2.60, 18.28)	<0.001	161	1.23 (1.02, 1.47)	1.20 (0.98, 1.47)	0.08
6	43	1.93 (1.15, 3.24)	1.76 (1.00, 3.07)	0.05	419	0.97 (0.85, 1.10)	0.97 (0.83, 1.13)	0.68
7	145	1.00	1.00	-	965	1.00	1.00	-
8	184	1.28 (0.86, 1.90)	1.23 (0.82, 1.86)	0.31	782	0.85 (0.76, 0.96)	0.91 (0.80, 1.04)	0.16
≥9	17	1.45 (0.60, 3.49)	1.65 (0.67, 4.03)	0.28	130	1.07 (0.87, 1.32)	1.15 (0.91, 1.45)	0.23
per 1 hour increase		0.81 (0.68, 0.98)	0.82 (0.69, 0.99)	0.04		0.95 (0.91, 0.99)	0.97 (0.93, 1.02)	0.21

Abbreviations: N, number; HR, hazard ratio; CI, confidence interval.

[a] Adjusted for sex and TNM stage.

[b] Adjusted for sex, TNM stage, treatments, multiple primary cancer, family history of cancer, body mass index, education, and employment.

[c] *P*-value was for the fully adjusted model.

Supplementary Table 13. Associations of lifestyle factors with all-cause mortality risk in early-/late-onset cancer survivors with non-low body mass index

	Early-onset (<50 years old)				Late-onset (≥50 years old)			
	N	Minimally adjusted HR (95% CI) [a]	Fully adjusted HR (95% CI) [b]	*P* [c]	N	Minimally adjusted HR (95% CI) [a]	Fully adjusted HR (95% CI) [b]	*P* [c]
**Smoking**								
Never/Former	4,273	1.00	1.00	-	13,064	1.00	1.00	-
Current	351	1.01 (0.85, 1.22)	0.96 (0.80, 1.15)	0.65	1,538	1.01 (0.94, 1.09)	1.02 (0.95, 1.09)	0.59
**Alcohol**								
Never/Former	4,473	1.00	1.00	-	13,964	1.00	1.00	-
Current	151	0.96 (0.75, 1.23)	0.94 (0.73, 1.21)	0.63	638	1.16 (1.05, 1.28)	1.18 (1.07, 1.30)	0.001
**Physical activity, minutes/week**								
None (inactive)	1,895	1.00	1.00	-	6,222	1.00	1.00	-
1-149 (insufficiently active)	1,360	0.81 (0.71, 0.94)	0.82 (0.72, 0.95)	0.007	3,895	0.84 (0.80, 0.89)	0.86 (0.81, 0.90)	<0.001
≥150 (active)	1,369	0.85 (0.74, 0.98)	0.88 (0.76, 1.01)	0.06	4,485	0.74 (0.70, 0.78)	0.76 (0.72, 0.81)	<0.001
per 60 minutes increase		0.96 (0.93, 0.99)	0.97 (0.94, 1.00)	0.05		0.94 (0.93, 0.95)	0.95 (0.94, 0.96)	<0.001
**Sleep duration, hours/day**								
≤5	65	1.43 (0.92, 2.21)	1.30 (0.84, 2.03)	0.24	400	1.05 (0.92, 1.19)	1.08 (0.93, 1.24)	0.30
6	349	0.97 (0.78, 1.20)	0.91 (0.72, 1.13)	0.38	1,715	0.99 (0.92, 1.07)	0.95 (0.87, 1.03)	0.18
7	1,644	1.00	1.00	-	5,916	1.00	1.00	-
8	2,413	0.94 (0.83, 1.06)	0.93 (0.82, 1.05)	0.24	6,058	0.93 (0.88, 0.98)	0.94 (0.89, 0.99)	0.03
≥9	153	0.89 (0.65, 1.23)	0.91 (0.66, 1.26)	0.58	513	0.92 (0.81, 1.04)	0.87 (0.75, 1.00)	0.05
per 1 hour increase		0.94 (0.88, 1.01)	0.95 (0.89, 1.02)	0.16		0.97 (0.95, 0.99)	0.98 (0.95, 1.00)	0.04

Abbreviations: N, number; HR, hazard ratio; CI, confidence interval.

[a] Adjusted for sex and TNM stage.

[b] Adjusted for sex, TNM stage, treatments, multiple primary cancer, family history of cancer, body mass index, education, and employment.

[c] *P*-value was for the fully adjusted model.

Supplementary Table 14. Measures of 5-year survival outcomes using Kaplan-Meier estimator for the univariate association

	Early-onset (<50 years old)			Late-onset (≥50 years old)		
	5-year RMST (95% CI)	RMST difference (95% CI)	*P*	5-year RMST (95% CI)	RMST difference (95% CI)	*P*
**All**	4.29 (4.25, 4.33)	-	-	3.45 (3.42, 3.48)	-	-
**Smoking**						
Never/Former	4.34 (4.30, 4.38)	-	-	3.49 (3.46, 3.52)	-	-
Current	3.67 (3.48, 3.85)	-0.67 (-0.86, -0.48)	<0.001	3.10 (3.01, 3.19)	-0.39 (-0.49, -0.30)	<0.001
**Alcohol**						
Never/Former	4.31 (4.27, 4.35)	-	-	3.47 (3.44, 3.50)	-	-
Current	3.55 (3.27, 3.83)	-0.76 (-1.04, -0.48)	<0.001	2.99 (2.85, 3.13)	-0.48 (-0.62, -0.33)	<0.001
**Physical activity, minutes/week**						
None (inactive)	4.12 (4.06, 4.19)	-	-	3.13 (3.09, 3.17)	-	-
1-149 (insufficiently active)	4.38 (4.31, 4.45)	0.25 (0.16, 0.35)	<0.001	3.57 (3.52, 3.63)	0.45 (0.38, 0.51)	<0.001
≥150 (active)	4.42 (4.35, 4.48)	0.29 (0.20, 0.39)	<0.001	3.81 (3.77, 3.86)	0.69 (0.62, 0.75)	<0.001
**Sleep duration, hours/day**						
≤5	3.82 (3.42, 4.23)	-0.43 (-0.84, -0.02)	0.04	2.98 (2.82, 3.14)	-0.44 (-0.61, -0.28)	<0.001
6	4.14 (3.99, 4.30)	-0.11 (-0.28, 0.06)	0.20	3.34 (3.26, 3.42)	-0.08 (-0.17, 0.01)	0.09
7	4.25 (4.19, 4.32)	-	-	3.42 (3.38, 3.46)	-	-
8	4.34 (4.28, 4.39)	0.09 (-0.001, 0.17)	0.05	3.55 (3.51, 3.59)	0.13 (0.07, 0.19)	<0.001
≥9	4.34 (4.14, 4.55)	0.09 (-0.13, 0.31)	0.42	3.47 (3.32, 3.61)	0.05 (-0.10, 0.20)	0.53

Abbreviations: RMST, restricted mean survival time; CI, confidence interval.
